# Editorial: Novel Molecular Mechanisms and Innovative Therapeutic Approaches for Age-Associated Diseases

**DOI:** 10.3389/fmolb.2022.922821

**Published:** 2022-05-18

**Authors:** Leming Sun, Zhen Fan, Zhe-Sheng Chen

**Affiliations:** ^1^ Key Laboratory of Space Bioscience & Biotechnology, School of Life Sciences, Northwestern Polytechnical University, Xi’an, China; ^2^ Department of Polymeric Materials, School of Materials Science and Engineering, Tongji University, Shanghai, China; ^3^ College of Pharmacy and Health Sciences, St. John’s University, Queens, NY, United States

**Keywords:** age-related diseases, molecular mechanisms, target therapy, therapeutic biotechnology, functional genomics

Aging is a risk factor for various chronic diseases, composed of but not limited to neurological, cardiovascular, metabolic, and autoimmune conditions, as well as disorders and even many types of cancers. It is challenging to conduct in-depth research of the molecular mechanisms associated specifically with human age-related diseases, and how internal and external factors are regulating these processes. For these reasons, research has focused on finding innovative therapies that increase the specificity of the treatment and reduce their drawbacks. This Research Topic on “N*ovel Molecular Mechanisms and Innovative Therapeutic Approaches for Age-Associated Diseases”* collects original and review articles to address current knowledge and progresses being made in the molecular mechanisms associated with age-related diseases, and innovative pharmaceutical experiments that are being developed.

This Research Topic described some high-throughput methods (transcriptome, metabolome, proteome, methylome, etc.) for examining age-related molecular signatures. Liu et al. offered insights into the heterogeneity of osteoarthritis, which provided in-depth understanding of the transcriptomic diversities within synovial tissue. This transcriptional heterogeneity may improve an understanding on osteoarthritis pathogenesis and provide potential molecular therapeutic targets for osteoarthritis. Zhao et al. investigated the aging-based diagnostic gene signature and molecular subtypes with diverse immune infiltrations in atherosclerosis. Huang et al. did comprehensive characterization of ageing-relevant subtypes associated with different tumorigenesis and tumor microenvironment in prostate cancer. The authors proposed that ageing-relevant molecular subtypes and gene signature might be of great significance to determine clinical outcomes and tumor microenvironment features as well as immunotherapeutic responses in prostate cancer. Song et al. identified reliable gene signatures through combination strategies of diverse feature selection methods, which facilitated the early detection of ischemic cardiomyopathy and revealed the underlying mechanisms. Liu et al. developed of a toll-like receptor (TLR)-based gene signature that can predict prognosis, tumor microenvironment, and chemotherapy response for hepatocellular carcinoma. They also proposed that this TLR-based gene signature might assist clinicians to select personalized therapy programs for HCC patients.

This Research Topic also discussed novel mechanistic insights and targeted therapies for a personalized treatment against various age-related diseases. Lin et al. summarized the role of macrophage phenotypic diversity in the progression of the dynamic atherosclerotic plaque, and the possibility of treating atherosclerosis by targeting macrophage microenvironment. Wang et al. reviewed and concluded the AMPK as a potential therapeutic target for intervertebral disc degeneration. The role of sonic hedgehog pathway in the development of the central nervous system and aging-related neurodegenerative diseases was discussed by Yang et al. You at al. investigated the influence of anemia on postoperative cognitive function in patients undergo hysteromyoma surgery. The results from He et al. showed that secoisolariciresinol diglucoside (SDG) can significantly increase mitochondrial DNA copy number and slow down the process of telomere shortening. The authors also indicated that SDG could improve ovarian reserve by inhibiting oxidative stress. Qin et al. discovered that rational design and synthesis of 3-morpholine linked aromatic-imino-1H-indoles as novel Kv1.5 channel inhibitors sharing vasodilation effects. Du et al. found that the 4-methoxydalbergione could inhibit bladder cancer cell growth through inducing autophagy and inhibiting Akt/ERK signaling pathway. Wu et al. suggested that the sevoflurane could alleviate myocardial ischemia reperfusion injury *via* inhibiting P2X7-NLRP3 mediated pyroptosis. Jia et al. demonstrated that circulating LBX2-AS1 could be an underlying diagnostic marker in multiple myeloma (MM). Targeting LBX2-AS1 suppressed tumor progression by affecting mRNA stability of LBX2 in MM. Hence, LBX2-AS1 could be a novel therapeutic marker against MM. Lan et al. uncovered that the olfactory impairment could be an early indicator to guide early intervention for postoperative cognitive dysfunction.

New formulations or new therapeutic molecules useful for increasing anti-aging effectiveness and reducing toxicity are illustrated here. The atheroprotective effects and molecular mechanism of berberine were discussed by Xing et al. Lu et al. reviewed the vitamin D and lipid profiles in postmenopausal women and concluded that the vitamin D administration in postmenopausal women could decrease the concentrations of triacylglycerol, and HDL-cholesterol, but have no effects on LDL-cholesterol and total cholesterol. Xu et al. summarized the recent progress in the functions and mechanisms of newly discovered circular RNAs in intervertebral disc degeneration. Zheng et al. stated that ginkgo biloba extract 80 could have cardioprotective properties through the activation of AKT/GSK3β/β-Catenin signaling pathway. Yin et al. investigated the cordyceps militaris-derived polysaccharide CM1 and demonstrated that it could alleviate atherosclerosis in LDLR (−/−) mice by improving hyperlipidemia. Zhou et al. revealed that propofol could ameliorate ox-LDL-induced endothelial damage by enhancing autophagy through PI3K/Akt/m-TOR pathway, which might offer a novel therapeutic strategy in atherosclerosis. A type I collagen-targeted MR imaging probe for staging fibrosis in Crohn’s disease was conducted by Li et al. Their results demonstrates that targeted MRI probe (EP-3533) supplies a better enhanced effect compared to Gd-DTPA and could be a promising method to evaluate the progression and monitor the therapeutic response of bowel fibrosis. Yang et al. found that the sinomenine could suppress development of hepatocellular carcinoma cells through inhibiting MARCH1 and AMPK/STAT3 signaling pathways. This study provides a new support for SIN as a clinical anticancer drug and illustrates that targeting MARCH1 could be a novel treatment strategy in developing anticancer therapeutics.

Finally, some genes and proteins involved in aging or anti-aging as well as substances that inhibit or rejuvenate aging are also presented in this Research Topic. Wang et al. found that the aging-related gene signature was in relation to tumor immunity and stromal activation in rectal cancer, which might predict survival outcomes and immuno- and chemotherapy benefits. Li et al. stated that the miR-330–5p in small extracellular vesicles derived from plastrum testudinis-preconditioned bone mesenchymal stem cells could attenuate osteogenesis by modulating Wnt/β-Catenin signaling. Zeng et al. observed that knockdown of miR-615-5p reversed the suppression of circRNA_100146 silence on the proliferation and invasion of prostate cancer cells. The authors also stated that the tumor growth was also suppressed by silencing circRNA_100146 *in vivo*. Lu et al. identified two osteoarthritis-specific markers HTR2B and SLC5A3 and their knockdown ameliorated apoptosis and inflammation of Osteoarthritis synovial cells. Zhang et al. also discovered that mitomycin C could inhibit esophageal fibrosis by regulating cell apoptosis and autophagy *via* lncRNA-ATB and miR-200b.

We believe that researchers could find this Research Topic to be a useful Research Topic of articles on novel molecular mechanisms and innovative therapeutic approaches for age-associated diseases.

